# Blood Group Substances as Potential Therapeutic Agents for the Prevention and Treatment of Infection with Noroviruses Proving Novel Binding Patterns in Human Tissues

**DOI:** 10.1371/journal.pone.0089071

**Published:** 2014-02-18

**Authors:** Shin Yazawa, Takehiko Yokobori, Gen Ueta, Munenori Ide, Bolag Altan, Aksara Thongprachum, Toyo Nishimura, Tamiko Nakajima, Yoshihiko Kominato, Takayuki Asao, Abby R. Saniabadi, Kiyoshi Furukawa, Hiroyuki Kuwano, Jacques Le Pendu, Hiroshi Ushijima

**Affiliations:** 1 Department of General Surgical Science, Gunma University Graduate School of Medicine, Maebashi, Japan; 2 Tokushima Research Institute, Otsuka Pharmaceutical Co. Ltd., Tokushima, Japan; 3 Laboratory of Glycobiology, Department of Bioengineering, Nagaoka University of Technology, Nagaoka, Japan; 4 Department of Diagnostic Pathology, Gunma University Graduate School of Medicine, Maebashi, Japan; 5 Department of Developmental Medical Sciences, School of International Health, Graduate School of Medicine, The University of Tokyo, Tokyo, Japan; 6 Division of Microbiology, Department of Pathology and Microbiology, Nihon University School of Medicine, Tokyo, Japan; 7 Department of Legal Medicine, Gunma University Graduate School of Medicine, Maebashi, Japan; 8 JIMRO Co., Ltd., Tokyo, Japan; 9 Inserm, UMR892; CNRS, UMR 6299; University of Nantes, Nantes, France; Okayama University, Japan

## Abstract

Blood group-related glycans determining ABO and Lewis blood groups are known to function as attachment factors for most of the norovirus (NoV) strains. To identify binding specificity of each NoV, recombinant norovirus-like particles (VLPs) and human saliva samples with different ABO, Lewis phenotypes and secretor status have been commonly applied. When binding specificities of VLPs prepared from 16 different genotypes of NoVs in GI and GII genogroups were characterized in samples of human gastric mucosa compared to human saliva based on blood group phenotypes, considerable differences were observed for several strains. Novel binding specificities determined by an ELISA using preparations from human gastric mucosa were also ascertained by immunohistochemical analyses using human jejunal mucosa, widely believed to be susceptible to NoV infection. Further, A, B and O(H) blood group substances prepared from porcine and squid tissues were found to be effective for preventing ABO blood group-specific binding of VLPs to both saliva and mucosa samples. Therefore, these blood group substances might have potential for the prevention and treatment of NoV infection.

## Introduction

Noroviruses (NoVs) are a group of single-stranded, positive sense RNA viruses constituting one of the six genera of the *Caliciviridae* family, and are known to be the predominant cause of non-bacterial acute gastroenteritis in humans worldwide [1]–[6]. They are classified into five genogroups (GI–GV) with a high genetic diversity and three of them (GI, GII and GIV) infect humans, which are grouped further into at least 15 (GI.1–GI.15) and 21 (GII.1–GII.21) genotypes [6]. Since the discovery of the prototype Norwalk virus (NV), later designated as GI.1, at Norwalk City, Ohio, U.S.A. in 1972 as the virus causing acute gastroenteritis [7], studies on NoVs have been mainly conducted based on molecular epidemiology and genetics as well as serology [8]–[9].

The establishment of recombinant virus-like particles (VLPs) from insect cell culture system using a recombinant baculovirus, which appeared to be indistinguishable from wild-type virus [10] contributed to significant progress not only in immunology of NoVs but also in understanding the mechanism of infection with NoVs. Noticeably, evidence supporting the association of infectious profiles with blood types of hosts was obtained from volunteer challenge studies first performed with NV (GI.1) [11]–[13] and more recently with GII.4 [14].

Early studies on binding specificity of the VLP from NV (GI.1) were conducted using a panel of human saliva samples whose ABO, Lewis blood group phenotypes and secretor status had been identified [15], [16]. In order to investigate ligand specificity of further identified NoVs and host-susceptibility factors for infection, it has been widely examined whether their recombinant VLPs could react with panels of human saliva samples, chemically synthesized oligosaccharides, human milk and epithelial cells of porcine gastrointestinal tissues [5], [17]–[23]. In addition, pathogenesis of NoVs infection has been investigated in 23 jejunal biopsy tissues from infected volunteers [24]. However, because of a lack of animal model and a culture system of infected cells, details of the mechanism for NoV infection are still unclear [9], [25], [26] which causes to hamper the development of efficient treatments.

In this study, VLPs prepared from 16 different genotypes of NoVs in GI and GII genogroups were examined for their binding specificities by an ELISA using not only a panel of human saliva samples but also preparations from human gastric mucosa (HGM) with different blood group phenotypes and secretor statuses. At the same time, resected mucosa samples from human jejunum, widely believed to be susceptible to NoV infection [27] along with the proximal small intestine [28], [29], were, for the first time, examined to demonstrate their binding profiles immunohistochemically with the knowledge of their blood types. Further, a novel treatment strategy against infection of NoVs was also investigated with A, B and O(H) blood group substances prepared from food ingredients [30].

## Materials and Methods

### Reagents

Anti-A, anti-B, anti-Le^a^ and anti-Le^b^ mouse monoclonal antibodies were obtained from Ortho Clinical Diagnosis (Rochester, NY) and biotinylated *Ulex* lectin was from Seikagaku (Tokyo, Japan). Skim milk, bovine serum albumin (BSA), L-fucose, Tween 20 and sodium metaperiodate were purchased from Sigma (St. Louis, MO). *Aleuria aurantia* lectin (AAL) immobilized Sepharose gel was prepared as described previously [31]. α1,3*N*-acetylgalactosaminidase from *Clostridium tertium* A [32] and α1,3galactosidase from *Clostridium sporogenesis* maebashi [33] and α1,2fucosidase from *Bacillus fulminans* [34] were prepared as described previously. α1,3/4Fucosidase from *Streptomyces* sp.142 was obtained from Takara Bio Inc. (Otsu, Japan) and β1,3galactosidase from *Xanthomonas manihotis* was obtained from New England BioLabs Inc. (Ipswich, MA). YB-3 antibody recognizing Fucα1,2Galβ linkages was prepared and purified as described previously [35]–[37]. Chemically synthesized oligosaccharides, such as Fucα1,2Galβ, Fucα1,2Galβ1,3GlcNAcβ, Fucα1,2Galβ1,4GlcNAcβ, Fucα1,2Galβ1,3GalNAcα, Fucα1,2Galβ1,3GalNAcβ, GalNAcα1,3[Fucα1,2]Galβ, Galα1,3[Fucα1,2]Galβ, Fucα1,2Galβ1,3[Fucα1,4]GlcNAcβ, Fucα1,2Galβ1,4[Fucα1,3]GlcNAcβ attached to bovine serum albumin (BSA) (Syntagens) and Fucα1,2Galβ attached to silica beads (Synsorb) were obtained from Chembiomed, Edmonton, Canada. HRP-labeled anti-mouse IgM and IgG and the ABC kit were obtained from Vectastain (Vector Laboratories, Burlingame, CA). HRP-labeled anti-rabbit IgG was from Jakson ImmunoResearch Lbs. Inc. (West Grove, PA). Histofine Simple Stain MAX PO was from Nichirei (Tokyo, Japan).

Human jejunal tissues (n = 9) were obtained from patients with gastric cancer who were admitted to Gunma University Hospital (Gunma, Japan). Human gastric mucosa (HGM) samples (n = 39) were also obtained from forensic autopsies. This study was approved by the Ethics Committee of Gunma University, and all the tissues were obtained along with the guideline for informed consent and approval from the Ethics Committee of Gunma University. Written informed consent was obtained from all the donors.

Porcine stomach tissues were purchased from Gunma Meat Wholesale Market Co. Ltd. (Gunma, Japan) and their gastric mucosa (PGM) typed as A^+^H^+^ and A^−^H^+^ were prepared and purified as described below. Japanese flying squids (*Todarodes pacificus*) were purchased from a local fisher market. Human saliva samples (n = 33) were also obtained from healthy volunteers. Human milk was obtained from an individual with OLe(a−b+)sec., and was prepared as described previously [38].

### Binding specificity of NoV to human saliva and gastric mucosa samples

Recombinant VLPs were expressed by the baculovirus expression system and were prepared as described previously [39], and purified VLPs were examined for particle formation by electron microscopy [40]. VLPs from sixteen different NoVs used in this study were as follows (strain and sequence accession number); GI.1 (4656, EF547392), GI.3 (3634, EF547393), GI.4 (2876, EF547394), GI.8 (3006, EF547395), GI.11 (2258, EF547396), GII.1 (3101, EF547397), GII.2 (2840, EF547398), GII.3 (3229, EF547399), GII.4 (1207, DQ975270), GII.5 (3611, EF5473400), GII.6 (3612, EF5473401), GII.7 (419, EF5473402), GII.12 (2087, EF5473403), GII.13 (3385, EF5473404), GII.14 (2468, EF5473405), and GII.15 (3625, EF5473406).

Rabbit polyclonal antibodies against aforementioned VLPs were as follows, that were kindly provided by Immuno Probe Co. Ltd. (Saitama, Japan); anti-GI.1 (Lot 71203), anti-GI.11 (Lot 71203), anti-GII.2 (Lot 71203), anti-GII.3 (Lot 71121), anti-GII.4 (Lot 71130), anti-GII.5 (Lot 71130), anti-GII.7 (Lot 81011), anti-GII.12 (Lot 81012) and anti-GII.15 (Lot 81013). Mouse monoclonal antibodies recognizing epitopes for multiple VLPs were prepared as described previously; MAb14-1 for GI.3, 4, 8 and 11, GII.1, 2, 3, 4, 5, 6 and 7 and GII.12, 13, 14 and 15 [37]. MAb12-12 for GI.3, 4 and 8 and MAb7-4 for GII.1, 14 and 15 were also prepared and kindly provided by Immuno Probe Co. Ltd.

Binding specificity of each NoV was examined by means of an ELISA using their VLPs and samples from saliva and HGM as described previously [15] with a slight modification. One hundred µl of saliva samples, diluted at 1∶500 with 0.1 M carbohydrate buffer (pH 9.5) or one hundred µl of HGM prepared as described below at 0.01% in saline was added into a NUNC immunoplate (Raskilde, Denmark) and incubated at 37°C overnight. After washing with 250 µl of 0.02 M phosphate buffered saline (pH 7.0, PBS) containing 0.05% Tween 20 (T-PBS) three times, each well of the plate was blocked with PBS containing 5% skim milk by an incubation for 1 h at 37°C, then the plate was washed with T-PBS three times. Fifty µl of each VLP at 1 µg/ml in 1% BSA/PBS was added to each well of the plate and incubated for 1 h at 37°C. After washing the plate with T-PBS, 100 µl of anti-VLP antibody corresponding to each VLP diluted at 1∶500 to 1∶1,000 in 5% skim milk/PBS was added to each well and the plate was incubated for 1 h at 37°C. After washing the plate with T-PBS again, HRP-conjugated anti-mouse or rabbit IgG antibody was added (100 µl/well) to the wells following by incubation for 1 h at 37°C. Color was developed by adding 200 µl of TMB substrate kit for peroxidase (Vector) and incubating for 5 min at room temperature following by adding 50 µl of 1N sulfuric acid. Reactions of each well were read at 450 nm. Each assay was conducted twice in duplicate.

### Histochemical analysis of VLPs bound to human tissues

Human jejunal tissues were resected and frozen soon after operation within an 1 h. Tissues were embedded in the Super Cryoembedding medium (SECTION-LAB, Hiroshima, Japan) and the cut surface of tissues was covered with an adhesive film (Cryofilm type IIC9, SECTION-LAB) and then frozen sections were prepared with the aid of microtome (Leica EM UC7, Wetzlar, Germany) according to the method described previously [41]. After fixing in 4% paraformaldehyde solution (Sigma), the sections were treated with 100% methanol containing 0.3% hydrogen peroxide and were pre-incubated in PBS containing 2% BSA overnight. After washing with PBS, sections were covered with VLP solution (∼5 µg/ml) in PBS containing 1% BSA and left at 37°C for 1 h. After washing three times with PBS, the sections were incubated with corresponding antibodies (mouse or rabbit) against each VLP (dilution of 1∶1,000) for 1 h at room temperature. After washing in PBS, the sections were covered with labeled anti-mouse or anti-rabbit IgG antibodies for 1 h at room temperature followed by treatment with the DAB solution (Nichirei Biosciences Inc., Tokyo, Japan) or Vectastain ABC kit according to the manufacturer's instructions. In the latter case, color was developed with a solution containing 0.02% 3-3′-diaminobenzidine tetrahydrochloride and 0.01% hydrogen peroxide in 0.05 M of ammonium acetate-citrate buffer, pH 6.0. The sections were then counterstained with hematoxylin, washed with distilled water, and mounted. Immunostaining of the sections with blood group-related antibodies or lectin was also conducted as described previously [37].

### Inhibition of VLP binding with blood group substances

Inhibition of VLP binding with blood group substances was performed by pre-incubation of VLP with an equal volume of 2% solution of partially purified blood group substance in PBS containing 1% BSA for 1 h at room temperature, or by treatment with the same 2% solution for 1h at room temperature after VLPs were incubated with respective saliva samples coated on the plate. These pre- or post-treatments of VLPs with blood group substances were followed by binding assay as aforementioned. Inhibition of VLPs binding was also investigated immunohistochemically using resected jejunal mucosa.

### Blood group phenotypes and secretor status

ABO and Lewis blood group phenotypes of individuals whose saliva, gastric and jejunal mucosa were used in this study were determined by a hemagglutination reaction of their red blood cells with corresponding antibodies and a lectin. The secretor status as well as antigen levels of A, B, O(H) and Le^a^ and Le^b^ were determined by a hemagglutination inhibition test using corresponding antibodies, a lectin such as anti-A, anti-B, *Ulex*, anti-Le^a^, anti-Le^b^ , and ABO, Le(a+b−), Le(a−b+) red cells, respectively to examine the presence of ABH and Lewis antigens in human saliva and mucosal samples and tissues from porcine and flying fish as described previously [31], [42].

### Preparations of blood group substances from human and animal tissues

Gastric mucosa samples from human (HGM) and porcine (PGM) were homogenized with PBS and the extract was centrifuged at 10,000×g for 20 min. The supernatant was digested with pepsin and then partially purified substances were obtained with a phenol extraction method [43], [44] in which blood group active glycoproteins were mainly separated from proteins by 95% phenol extraction, and the phenol insoluble materials after recovering in the water and an ammonium sulfate fractionation were precipitated in 50–60% ethanol and obtained as purified glycoproteins. The liver from a flying squid was homogenized with PBS and the extract was centrifuged at 10,000×g for 20 min. Ethanol was added to the supernatant up to 50% with stirring. After centrifugation, the precipitates obtained were dissolved in a small amount of distilled water.

Since ABO(H) and Lewis blood group substances contain α-fucosyl residues as a part of the antigenic determinants, fucosylated antigens in the tissues extracts except HGM could be purified by means of the fucose-binding lectin, AAL immobilized on agarose gel as described previously [31], [45].

After dialysis against distilled water and lyophilization, 2% solution of each freeze-dried materials in PBS was applied onto an AAL-agarose column, and the absorbed materials were then eluted by adding PBS containing 20 mM L-fucose.

All the preparations were dialyzed against distilled water and then lyophilized. Blood group activities of these preparations were determined by means of hemagglutination inhibition tests as described above. Accordingly, PGMs were determined to possess commonly either A^+^H^+^ or A^-^H^+^ activity. Whereas, the presence of blood group B^+^H^+^ activity was demonstrated in the liver fraction of a flying squid (detailed results will be published elsewhere).

### Periodate oxidation and deglycosylation

To modify glycan structures of saliva samples to which each VLP bound, periodate oxidation and glycosidase treatments were conducted resulting in release of glycan moieties from the protein and deglycosylation of corresponding glycans, respectively. Accordingly, changes of blood group specificity were assayed after these treatments by means of hemagglutination inhibition test as above.

Saliva samples from A, B and OLe(a−b+) secretors were treated with 0.1 M sodium metaperiodate in 0.04 M sodium acetate buffer, pH 4.5 at 4°C overnight in the dark. The reaction was stopped by the addition of an equal volume of 0.2 M glycerol. Each sample was then dialyzed against distilled water and lyophilized. The dried materials were dissolved in PBS and used for ELISA. Blood group specific deglycosylations of saliva samples from ALe(a−b+), BLe(a−b+) and OLe(a−b−) secretor individuals were conducted by treatments with α1,3*N*-acetylgalactosaminidase (*C. teritium* A), α1,3galactosidase (*C. sporogenesis* maebashi) and α1,2fucosidase (*B. fulminans*) preparations, respectively, in 20 mM sodium phosphate, pH 7.0 at 37°C overnight. Saliva samples from OLe(a+b−) and OLe(a−b−) non-secretors were also treated with α1,3/4fucosidase (*S.* sp.142) and β1,3galactosidase (*X. manihotis*), respectively according to the manufacturers' instructions at 37°C overnight. After boiling for 2 min, the supernatant obtained by centrifugation at 10,000 g for 10 min was used for ELISA as described above.

## Results

### Binding of NoV-VLPs to saliva samples with different ABO and Lewis blood groups, and secretor status

**Figure 1:**
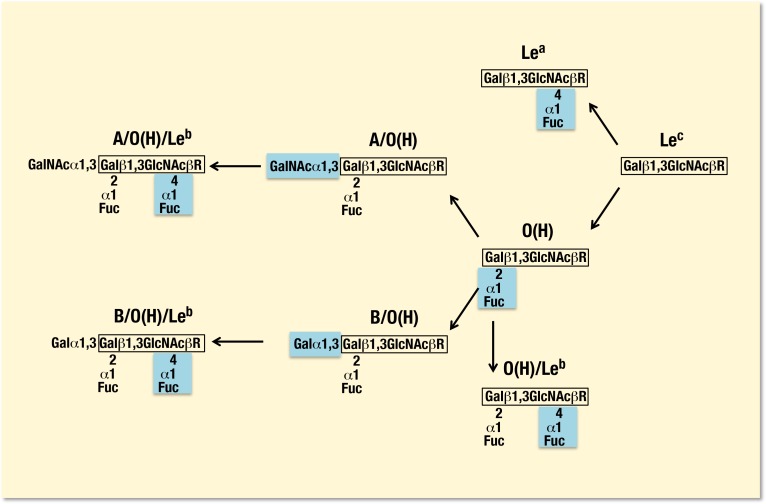
Immunological determinants of ABO and Lewis blood groups synthesized on the type 1 chain structure. Gal, D-galactose; GalNAc, *N*-acetyl-D-galactosamine; Fuc, L-fucose; GlcNAc, *N*-acetyl-D- glucosamine; R, a remainder of molecule. The terminal sugars screened were added through sequential actions of type-specific glycosyltransferases present in corresponding blood types of saliva and tissues (See the details in the Text).

As listed in Figure 1, ABO and Lewis blood group-related determinants are commonly synthesized sequentially on the type 1 structure from Le^c^ (Galβ1,3GlcNAcβR) to di-fucosylated A (A/O(H)/Le^b^, GalNAcα1,3[Fucα1,2]Galβ1,3[Fuc1,4]GlcNAcβR) and/or di-fucosylated B (B/O(H)/Le^b^, Galα1,3[Fucα1,2]Galβ1,3[Fucα1,4]GlcNAcβR) determinants in saliva and tissues. Accordingly, binding patterns of VLPs were classified in relation to reactivity to 8 determinants such as di-fucosylated A, B (A or B/O(H)/Le^b^ ) and di-fucosylated O(H) (O(H)/Le^b^, Fucα1,2Galβ1,3[Fucα1,4]GlcNAcβR), mono-fucosylated A (A/O(H), GalNAcα1,3[Fucα1,2]Galβ1,3GlcNAcβR), B (B/O(H), Galα1,3[Fucα1,2]Galβ1,3GlcNAcβR) and O(H) (Fucα1,2Galβ1,3GlcNAcβR), Le^a^ (Galβ1,3[Fucα1,4]GlcNAcβR) and Le^c^. In addition, unlike in red blood cells, Le^a^ as well as O(H) determinants are present to some extent with Le^b^ and A or B determinants in saliva and tissues of ALe(a−b+) and BLe(a−b+) secretor individuals which could be detected with the aid of corresponding antibodies [35], [44].

**Figure 2:**
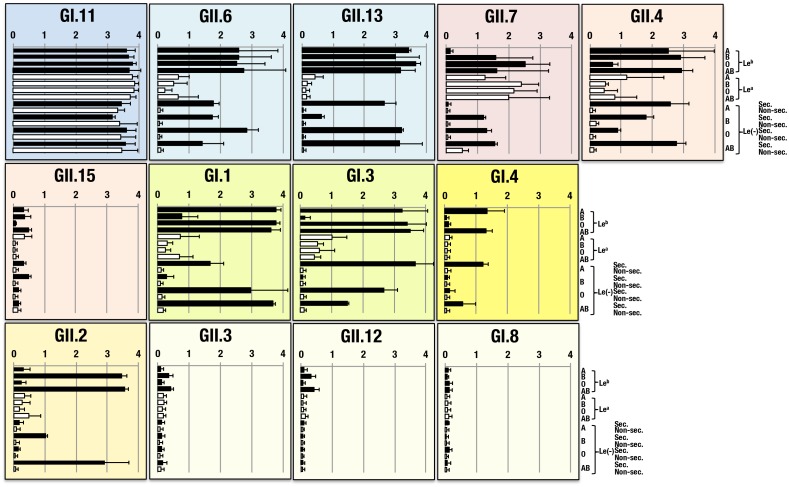
Binding assay of 13 NoV-VLPs to human saliva samples. Binding assays with human saliva (n = 32) from individuals with different ABO and Lewis phenotypes and secretor status were conducted using three different samples each from ABO(H) and Lewis-positive (Le^a+^ and Le^b+^) individuals and a single sample each from Lewis-negative (secretor and non-secretor) ones. Experiments of each sample were performed twice in duplicate and mean±SD values per individual blood types are given on the abscissa in the graph indicating optical density at 450 nm. At least eight different binding patterns were determined. See the details in the Text. Le^b^, Le(a−b+)sec.; Le^a^, Le(a+b−)non-sec.; Le(-), Le(a−b−); Sec., secretor; Non-sec., non-secretor.

**Figure 3:**
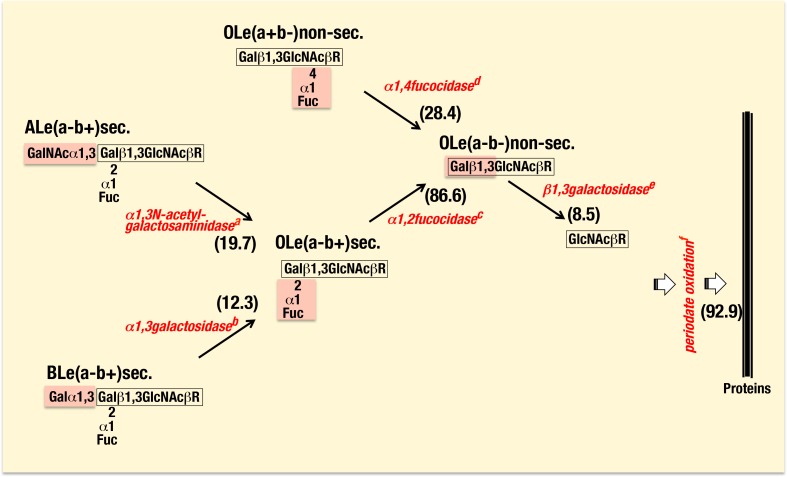
Releases of blood group specific glycans from saliva samples. Each group specific glycan was decomposed through the action of corresponding glycosidases and by periodate oxidation. Glycosidases treated were from ^a^ *Clostridium tertium* A, ^b^ *Clostridium sporogenesis* maebashi, ^c^ *Bacillus fulminans*, ^d^ *Streptomyces* sp.142, and ^e^ *Xanthomonas manihotis*. ^f^Periodate oxidation was conducted in saliva samples from ALe(a−b+)sec., BLe(a−b+)sec., OLe(a−b+)sec., OLe(a+b−)non-sec., and OLe(a−b−)non-sec. Numbers in the parenthesis indicate attenuation rations for binding with GI.11 after digestions of terminal glycans of respective saliva samples with corresponding glycosidases and periodate oxidation of saliva samples from A, B and O types, which were tentatively determined by subtractions of values obtained from ELISA for binding activity of untreated and treated samples (see the details in the Text).

Sixteen VLPs belonging to GI and GII genogroups were examined to determine their binding specificities using an ELISA plate coated with a panel of saliva samples from various ABO, Lewis blood group phenotypes and secretor status (Figure 2). Binding patterns of each VLP were deduced from the determinants (Figure 1) present in the saliva samples, and eight different binding patterns were determined resulting from assays with 12 VLPs (Table 1). Seven of the 16 VLPs showed very weak (GII.3, GII.12 and GII.15) or no clear (GI.8, GII.1, GII.5 and GII.14) binding pattern with all the saliva samples tested. Results from these VLPs except GI.8 VLP (GII.1, GII.5 and GII.14) were, therefore, not shown in Figure 2. Blood group activities of saliva samples used in this study were determined together with their ABO and Lewis blood group phenotypes and secretor status (Table 2). The VLP from GI.11 bound to all in a panel of saliva samples with different A, B, O and Lewis blood group phenotypes irrespective of their secretor statuses. No significant difference was observed between blood group phenotypes and secretor status. However, a significant decrease of binding was determined between this VLP and a saliva sample from an O_h_ Bombay individual (OD_450_ = 0.619±0.147, *P*<0.05) in which no A, B O(H) or Le^b^ antigen but only a small amount of Le^a^ antigen was secreted. More than 90% (92.857±3.487%, attenuation ratio) of the binding observed in GI.11 with saliva samples from A, B and OLe(a−b+) secretors was abolished after removal of glycans attached to their saliva samples by means of periodate oxidation (Figure 3). For further characterization of binding specificity, GI.11 VLP was examined using a series of saliva samples, following modification of their antigenic determinants by means of digestions with glycosidases. When saliva samples from A, B and OLe(a−b+) secretors were treated with α1,3*N*-acetylgalactosaminidase (*C. teritium* A), α1,3galactosidase (*C. sporogenesis* maebashi) and α1,2fucosidase (*B. fulminans*), respectively, their antigenic determinants were specifically destroyed resulting in decreases of GI.11 binding to each sample and attenuation ratios were tentatively calculated as 19.7, 12.3 and 86.6%, respectively. Likewise, treatments of saliva samples from OLe(a+b−) and OLe(a−b−) non-secretors with α1,3/4fucosidase and β1,3galactosidase, respectively, also caused diminished binding of GI.11 to them and their attenuation ratios were 28.4 and 8.5%, respectively. Therefore, binding of GI.11 to saliva involved type-specific glycans, and the data indicate that GI.11 has the broadest binding pattern of reactivity with not only di-fucosylated and mono-fucosylated A, B and O(H) but also Le^a^ and Le^c^ determinants as described below. VLPs from 3 NoVs were found to show rather broad binding patterns reacting with di-fucosylated and mono-fucosylated A, B and O(H) (GII.6, GII.13) and B, O(H) and Le^a^ (GII.7) determinants, respectively. Of note, none of these strains could bind significantly to the saliva samples from non-secretor or Lewis negative individuals, indicating lack of recognition in absence of an α1, 2fucosyl residue (GII.6, GII.13) or both α1, 2 and α1, 4fucosyl residues (GII.7) on the Le^c^ determinant (*P*<0.01). VLPs from the other NoVs seemed to show a gradually narrower reactivity to the same saliva samples. The VLPs from GII.4 and GII.15, and from GI.1 and GI.3 reacted significantly (*P*<0.01) with di-fucosylated and mono-fucosylated A, B and di-fucosylated and mono-fucosylated A, O(H) determinants over O(H) and B determinants, respectively. Further, four VLPs showed very specific binding patterns to di-fucosylated and mono-fucosylated A (GI.4), di-fucosylated and mono-fucosylated B (GII.2) (*P*<0.01), and di-fucosylated B (GII.3, GII.12) (*P*<0.05) determinants over the other ones, respectively.

**Table 1:** Probable binding specificities of NoV-VLPs examined in this study.

Glycan structures on the type 1 chain*	VLPs^#^
Di-fucosylated/mono-fucosylated A, B, O(H) and Le^a^, Le^c^	GI.11
Di-fucosylated/mono-fucosylated A, B, O(H)	GII.6, GII.13
Di-fucosylated/mono-fucosylated B, O(H) and Le^a^	GII.7
Di-fucosylated/mono-fucosylated A, B	GII.4, GII.15
Di-fucosylated/mono-fucosylated A, O(H)	GI.1, GI.3
Di-fucosylated/mono-fucosylated A	GI.4
Di-fucosylated/mono-fucosylated B	GII.2
Di-fucosylated B	GII.3, GII.12

**Figure 4:**
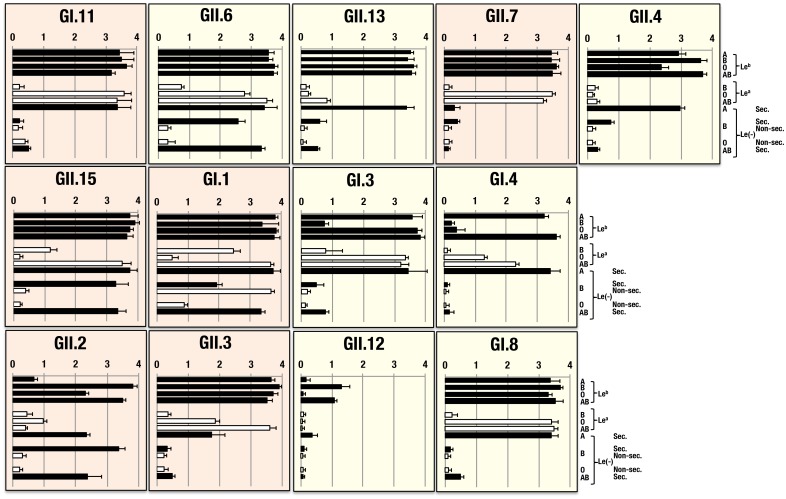
Binding assay of 13 NoV-VLPs to human gastric mucosa samples. Binding assays with human gastric mucosa samples from individuals with different ABO, Lewis phenotypes and secretor status were conducted using twelve samples. No samples of 4 blood types (ALe(a+b−)non-sec., ALe(a−b−)non-sec., OLe(a−b−)sec. or ABLe(a−b−)non-sec.) was available. Experiments of each sample were performed twice in duplicate and mean ± SD values per individual blood types are given on the abscissa in the graph indicating optical density at 450 nm. HGM samples were not from the same individuals as saliva ones. At least 6 different binding patterns (GI.11, GII.7, GII.15, GI.1, GII.2 and GII.3) were observed from saliva samples (Figure 2).

Binding specificities were grouped based on the results from ELISA using a panel of saliva samples (Fig. 2). *See the detailed structures in Fig. 1.^ #^Among 16 VLPs at least 6 VLPs (GI.11, GII.7, GII.15, GI.1, GII.2 and GII.3) showed different binding specificities of blood group-related glycans from those in gastric mucosa samples (Fig. 4). Four VLPs (GI.8, GII.1, GII.5 and GII.14) were not listed in this table (see the details in the Text).

**Table 2:** Hemagglutination inhibition titers of saliva and gastric mucosa samples against anti-ABH and Lewis antibodies and a lectin.

	HAI titers (1:n)*									
	Saliva					Gastric mucosa				
**Blood type**	**anti-A**	**anti-B**	**anti-H**	**anti-Le** ^a^	**anti-Le^b^**	**anti-A**	**anti-B**	**anti-H**	**anti-Le^a^**	**anti-Le^b^**
ALe(a−b+)sec.	32–2,048	n.t.	2–64	<2	64–2,048	2,560	n.t.	2,560	1,280	20,480
BLe(a−b+)sec.	n.t.	16–128	<2–16	32–64	256–512	n.t.	20,480	1,280	160	640
OLe(a−b+)sec.	n.t.	n.t.	32–256	20	256–2,048	n.t.	n.t.	5,120	2,560	20,480
ABLe(a−b+)sec.	16–4,096	32–64	16–64	16–64	256–1,024	5,120	2,560	80	640	5,120
ALe(a+b−)non-sec.	<2–8	n.t.	<2	128–1,024	<2–128					
BLe(a+b−)non-sec.	n.t.	<2–2	<2	128–256	<2	n.t.	<10	<10	320	<10
OLe(a+b−)non-sec.	n.t.	n.t.	<2	256	<2	n.t.	n.t.	<10	640	<10
ABLe(a+b−)non-sec.	<2–128	<2	<2	1,024–4,096	<2–64	16	<10	<10	1,280	<10
ALe(a−b−)sec.	1,024	n.t.	n.t.	16	<2	20,480	n.t.	2,560	<10	<10
ALe(a−b−)non-sec.	8	n.t.	<2	8	2					
BLe(a−b−)sec.	n.t.	128	<2	<2	<2	n.t.	80	20	<10	<10
BLe(a−b−)non-sec.	n.t.	<2	<2	<2	2	n.t.	<10	<10	<10	<10
OLe(a−b−)sec.	n.t.	n.t.	128	<2	4					
OLe(a−b−)non-sec.	n.t.	n.t.	<2	4	2	n.t.	n.t.	<10	<10	<10
ABLe(a−b−)sec.	256	64	32	2	4	160	80	40	<10	<10
ABLe(a−b−)non-sec.	4	2	<2	2	2					

*Three samples were tested in each saliva from ABO(H), Lewis-positive individuals and a single sample was tested in each saliva from Lewis-negative individuals and in each gastric mucosa. sec., secretor; non-sec., non-secretor. n.t., not tested. HAI, hemagglutination inhibition.

Binding activities of VLPs to saliva samples seemed to depend on the amounts of A, B, O(H), Le^a^ and Le^b^ blood group substances secreted in saliva, which could be quantified by mean of hemagglutination inhibition titers obtained from hemagglutination inhibition tests using corresponding antibodies or lectin and red blood cells (Figure 2, Table 2). In addition, binding activities of VLPs from five NoVs (GII.7, GII.4, GII.15, GI.1 and GI.3) showed to be influenced more or less by the Le^a^ determinant in saliva samples since some degrees of positive reactions were detected with saliva samples from Le(a+b−) non-secretors irrespective of their ABO blood phenotypes.

### Binding of NoV-VLPs to preparations from human gastric mucosa with different ABO, Lewis blood group phenotypes and secretor status

Sixteen VLPs were also examined to determine their binding activities to HGMs with different blood groups and secretor status (Figure 4). When compared with the results obtained using saliva samples (Figure 2), together with increased complexities, it was also found that binding patterns were often different from those determined by saliva samples. For one VLP (GII.12), a perfectly congruent pattern was observed between saliva and HGM samples. For five additional VLPs (GII.6, GII.13, GII.4, GI.3 and GI.4), binding patterns between saliva and HGM samples were rather similar except that a marked higher reactivity was found among HGM samples. Inconsistent patterns between saliva and HGM samples were present at least in six of the 16 VLPs (GI.11, GII.7, GII.15, GI.1, GII.2 and GII.3). In some of these cases, no positive reactivity with HGM samples was found with saliva samples of the same ABO and Lewis blood phenotypes and secretor status. In others, it was the opposite. Binding patterns were then grouped corresponding to the blood group-related determinants synthesized on the type 1 chain structures, as described above (Figure 1). More than five different binding patterns in which most of the VLPs bound in common to di-fucosylated and mono-fucosylated A, B and O(H) determinants were observed. The VLP from GI.1 seemed to bind to all the HGMs possessing di-fucosylated and mono-fucosylated A, B, O(H), and Le^a^ and Le^c^ determinants except one HGM from OLe(a+b−) non-secretor. Whereas, VLPs from GII.2, GII.4 and GII.13 were observed to react with di-fucosylated and mono-fucosylated A, B and O(H) determinants in HGMs. Further, six other VLPs also showed the same binding specificity with some extent of Le^a^ reactivity added (GI.8, GII.6, GII.7 and GII.15) and with Le^a^ reactivity added and mono-fucosylated B determinant deleted (GI.11 and GII.3). The VLP from GI.4 seemed to show the same binding specificity as in a panel of saliva samples but with some extent of Le^a^ determinant added. In addition, no specific binding pattern was observed for three VLPs (GII.1, GII.5 and GII.14) with 12 different blood types of HGMs (data not shown in Figure 2).

**Figure 5:**
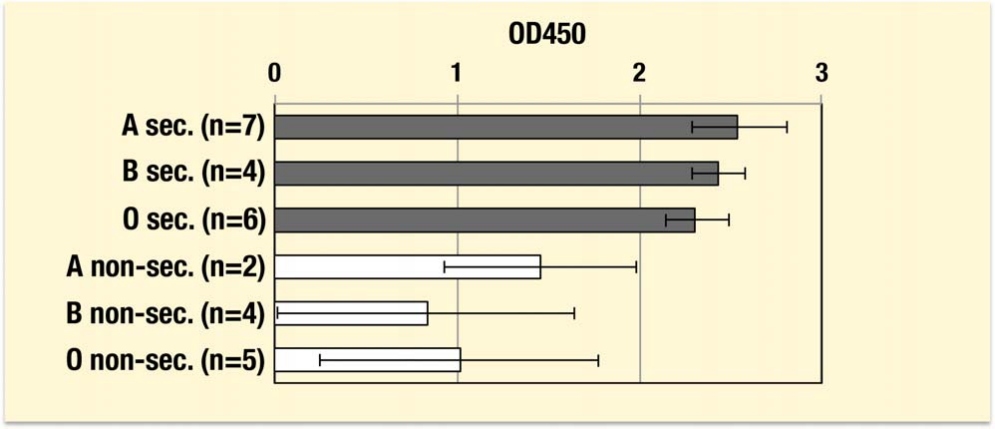
Binding assay of VLP from GI.1 to human gastric mucosa. Experiments were performed twice in duplicate and mean ± SD values obtained from indicated numbers of samples from individuals with different ABO phenotypes and secretor status. sec., secretor; non-sec., non-secretor.

The VLP from GI.1 showed aberrantly to react with HGMs irrespective of their ABO phenotypes, which was inconsistent with earlier reports and with the result using a panel of saliva samples in this study. To confirm these discrepancies, the GI.1 VLP was examined with increased numbers of HGM samples from different ABO groups and secretor status (Figure 5). It was determined that the GI.1 VLP was strongly reactive with HGMs from all the secretor individuals irrespective of their ABO blood groups, and a significant difference was observed between secretors and non-secretors from A, B and O blood type individuals (*P*<0.01) even though some of the HGMs from non-secretor individuals showed certain levels of the reactivity. The reactivity seemed to depend on the amounts of A, B and O(H) antigens present in their preparations which were determined by hemagglutination inhibition tests and on the dilution fold of the HGM preparations (data not shown).

### Inhibition of NoV-VLPs binding to human saliva samples with different blood group substances

**Figure 6:**
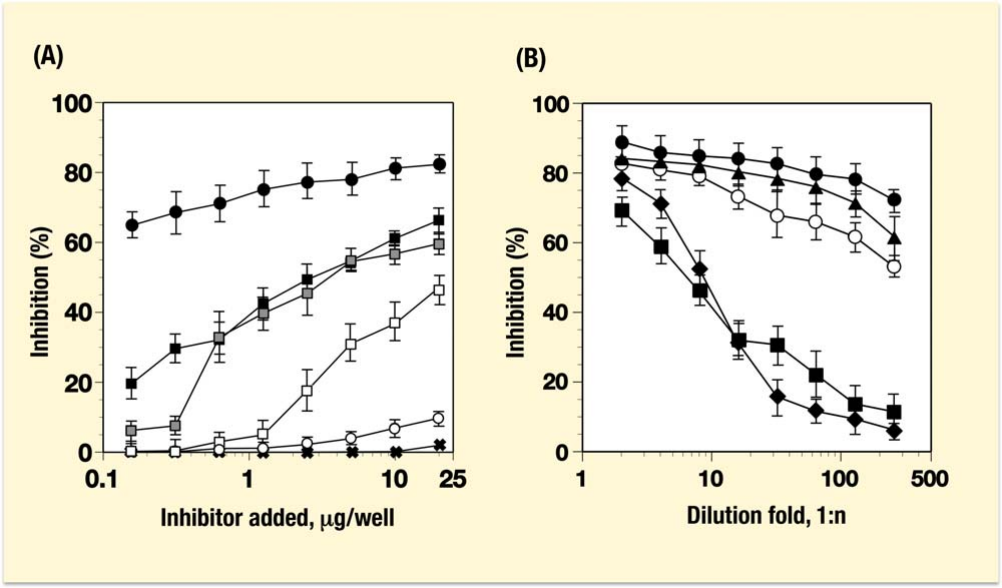
Inhibition of GI.1-VLP binding to saliva samples. (A) Binding of GI.1-VLP to a saliva sample from ALe^b^ secretor under the presence of HGMs and synthetic compounds. HGMs from ALe(a−b+)sec. (•), BLe(a−b+)sec. (▪), BLe(a−b−)sec. (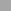), BLe(a−b−)non-sec. (□). Syntagen Y (○), and other Syntagens (⊤). Syntagen Y, Fucα1,2Galβ1,4[Fucα1,3]GlcNAcβ-BSA; other Syntagens containing A (GalNAcα1,3[Fucα1,2]Galβ), B (Galα1,3[Fucα1,2]Galβ), H type 1 (Fucα1,2Galβ1,3GlcNAcβ), H type 2 (Fucα1,2Galβ1,4GlcNAcβ), H type 3 (Fucα1,2Galβ1,3GalNAcα), H type 4 (Fucα1,2Galβ1,3GalNAcβ), Le^a^ (Galβ1,3[Fucα1,4]GlcNAcβ), Le^b^ (Fucα1,2Galβ1.3[Fucα1,4]GlcNAcβ), X (Galβ1,4[Fucα1,3]GlcNAcβ), T (Galβ1,3GalNAcα), Le^c^ (Galβ1,3GlcNAcβ) and LacNAc (Galβ1,4GlcNAcβ) attached to BSA. (B) Binding of GI.1 to a saliva sample from OLe^b^ secretor with serially diluted blood group substances. Concentrations of each substance were adjusted to obtain the same reactivity with YB-3 monoclonal antibody. PGM (H^+^) (•), human saliva (OLe(a−b+)sec.) (▴), PGM (H^+^A^+^) (○), human saliva (OLe(a−b−)sec.) (▪), and human milk (OLe(a−b+)sec.) (♦). See the details in the Text.

Inhibition of NoV-VLPs binding to saliva samples was also examined with the aid of blood group substances prepared from different sources. GI.1 VLP was pre-treated with HGM preparations from different blood types and a series of chemically synthesized blood group-related oligosaccharides attached to BSA (Syntagens) before the binding assay on a plate coated with the saliva from ALe(a−b+) secretor. Extremely strong inhibitions were detected by using the HGM preparation from ALe(a−b+) secretor over a wide rage of concentrations but only at higher concentrations of HGMs from BLe(a−b+) and BLe(a−b−) secretors, which was consistent with earlier reports indicating poor recognition of B blood type by the NV (GI.1) strain (Figure 6A). Chemically synthesized oligosaccharides including A, B and O(H) active tetrasaccharides attached to BSA were far less powerful inhibitors since only a weak activity was detected with the Le^Y^ (Fucα1,2Galβ1,4[Fucα1,3]GlcNAcβ) Syntagen at high concentrations.

Inhibition assay was also conducted on a plate coated with a saliva sample from OLe(a−b+) secretor using blood group substances prepared from different sources of secretor individuals, in which large molecules possessing O(H) blood group activity are commonly present (Figure 6B). Concentrations of each substance tested as inhibitors were adjusted to the same reactivity with the YB-3 monoclonal antibody specifically recognized with the H-active, Fucα1,2Galβ disaccharide structures irrespective of their backbone structures [36], [37]. Although the reactivity against Fucα1,2Galβ structures in them was adjusted before assay, five preparations from porcine gastric mucosa and human saliva and milk revealed different inhibiting activities over a wide rage of concentrations with a dose-dependency (Figure 4B). The PGM (A^−^H^+^) showed more potent activity than the PGM (A^+^H^+^) and the other blood group substances from human sources. Interestingly, saliva sample from OLe(a−b+) possessed an inhibiting activity more than thirty-times higher than that from OLe(a−b−), probably due to the presence of difucosylated O(H) substance in the former sample.

These results suggested that binding of GI.1 VLP to saliva samples, which was observed significantly both in A and O(H) blood types (Figure 2), was preferentially inhibited by blood group substances with the same blood types. In addition, PGM and human saliva and milk samples with an immunologically equivalent O(H) activity showed different inhibiting activities against GI.1 VLP binding to saliva sample from OLe(a−b+)sec.

### Blocking of VLP binding to saliva samples by means of blood group substances

**Figure 7:**
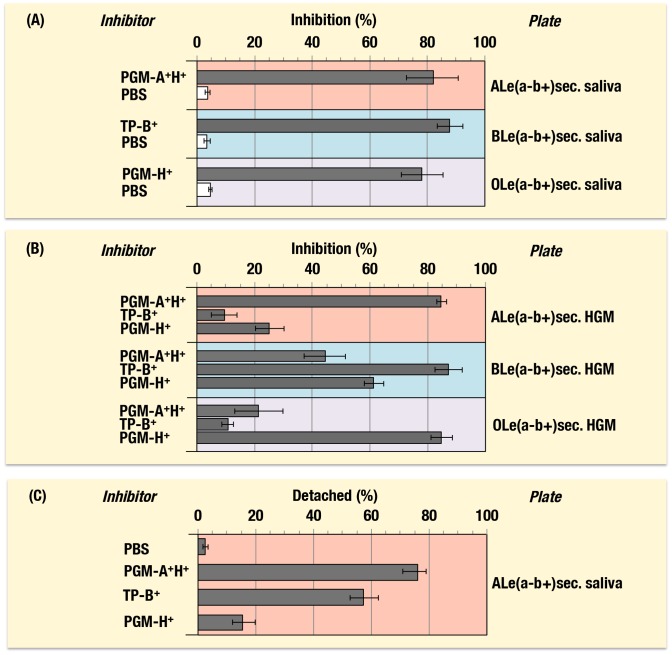
Blocking of type-specific binding of VLP with blood group substances. (A) Binding assays were performed using three plates coated with saliva samples from ALe(a−b+), BLe(a−b+) and OLe(a−b+), respectively. Prior to the assay, GI.1-VLP was incubated with blood group substances from PGM (A^+^H^+^), the flying squid liver (TP-B^+^) and PGM (A^−^H^+^), respectively. PBS, 0.01 M phosphate buffered saline (pH 7.0). (B) Binding assays were performed using three plates coated with HGM samples from ALe(a−b+), BLe(a−b+) and OLe(a−b+), respectively. Prior to the assay, GII.4-VLP was incubated with blood group substances from PGM (A^+^H^+^), the flying squid liver (TP-B^+^) and PGM (A^−^H^+^), respectively. (C) Binding assays were performed using a plate coated with a saliva sample from ALe(a−b+). After the GII.13-VLP bound to the plate, blood group substances from PGM (A^+^H^+^), the liver fraction (TP-B^+^) and PGM (A^−^H^+^) were added, respectively, prior to detection of bound VLP with anti-GII.13 antibody. PBS was added in the plate in place of blood group substances as a control. Experiments were performed twice in duplicated. Mean ± SD values are given on the abscissa in the graph indicating optical density at 450 nm. sec., secretor; non-sec., non-secretor.

Together with PGM preparations possessing blood group A (A^+^H^+^) and O(H) (A^−^H^+^) activities, respectively, blood group B active substance was prepared from the flying squid liver for the first time. Based on the above results indicating type specific inhibitors, blood group A, B and O(H) active substances were further examined for their abilities to block binding of VLP to saliva and HGM samples. As shown in Figure 2, the VLP from GI.1 binds strongly to both A and O(H) active saliva samples and weakly to B active ones. When GI.1 VLP was incubated with blood group substances from PGM (A^+^H^+^), flying squid liver (B^+^H^+^) and PGM (A^−^H^+^) prior to the binding assay using plates coated with saliva samples from A, B and OLe(a−b+) secretors, respectively, most of the VLP binding to respective plates were inhibited (Figure 7A). Without such pre-incubations of the VLP with corresponding substances (only with PBS), no significant inhibition was observed.

The VLP from GII.4 binds strongly to Le^b^ active HGM irrespective of their ABO phenotypes and saliva samples from A and BLe(a−b+)sec., but weakly to OLe(a−b+)sec. (Figure 2). When binding assays using GII.4 were conducted under the presence of the same A, B and O(H) blood group substances, type specific inhibitions were also clearly observed (Figure 7B). Accordingly, binding of the GII.4 to the plates coated with three types of HGM could be efficiently inhibited by corresponding types of blood group substances, respectively; A, B and O(H) blood group substances against binding of GII.4 to HGMs from A, B and OLe(a−b+), respectively. Inhibiting activities of B and O(H) substances in the A type HGM plate, A and O(H) active substances in the B type HGM plate, and A and B active substances in the O type HGM plate suggested, therefore, the occurrence of cross reactions of the GII.4 VLP with their co-existing H active sites.

As demonstrated above in ELISA with saliva and HGM preparations, the VLP from GII.13 binds strongly with all the samples from Le^b^ positive A, B and O individuals. When the A, B and O(H) active substances were added, respectively, to each well of the plate after the VLP from GII.13 bound to ALe(a−b+)sec. saliva that had been coated on the plate, most of the VLP were detached with PGM−A^+^H^+^ (Figure 7C). More than half and less than 20% of the VLP were also detached when the flying squid liver preparation (B^+^H^+^) and PGM (A^−^H^+^) were added to the same plate, respectively, while no detachment of the VLP was found by addition of PBS.

### Immunohistochemical detections of VLPs binding to human jejunal mucosa sections

**Figure 8:**
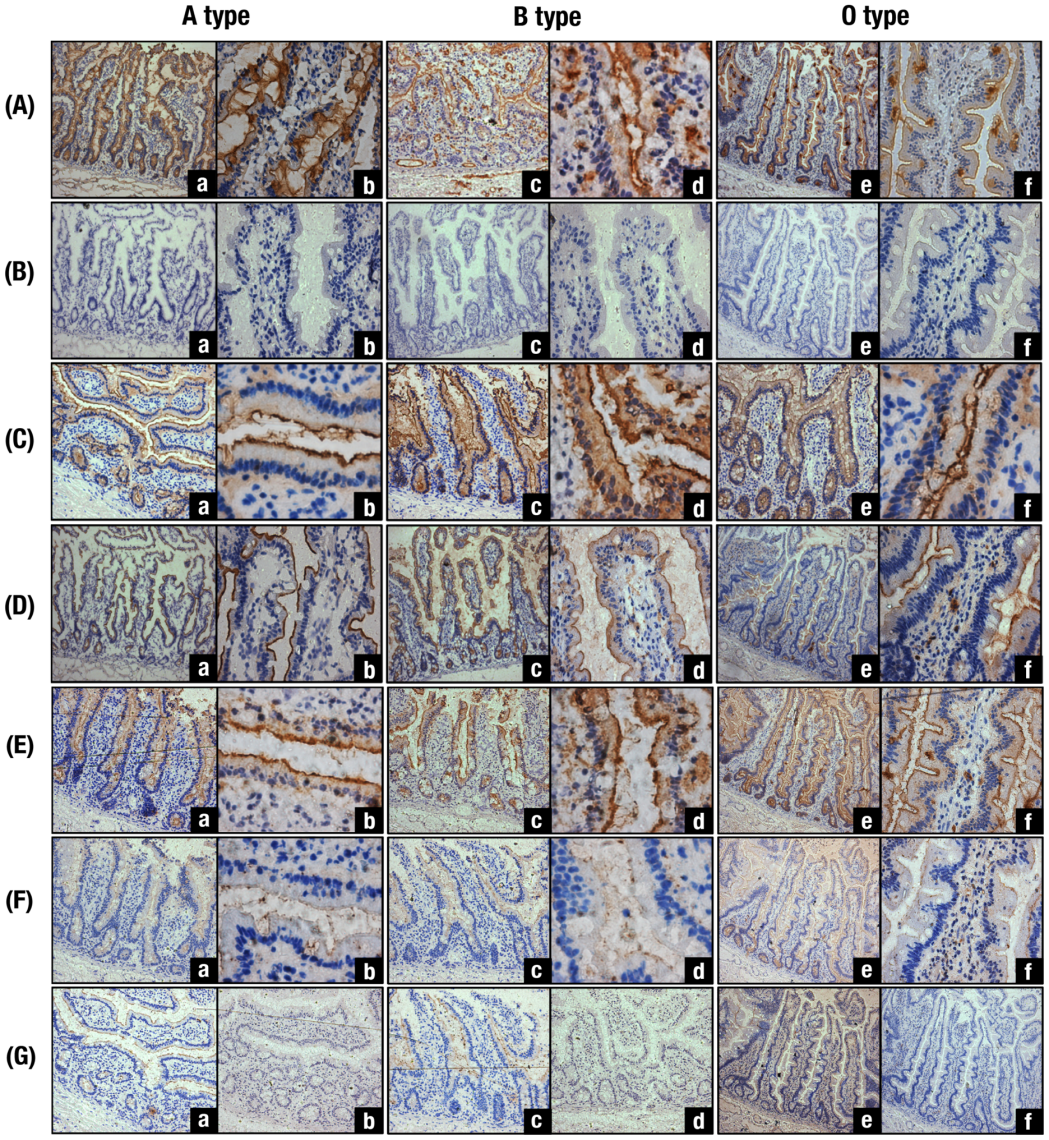
Binding assay of various VLPs to human jejunal mucosa sections from A, B and O blood types. (A) Sections were incubated with anti-A (a,b), anti-B (c,d) and biotinylated *Ulex* lectin (e,f). (B) Sections were incubated with PBS in place of anti-A, anti-B and *Ulex*. (C) Sections were incubated with GI.1 VLP and then with anti-GI.1 antibody. (D) Sections were incubated with GII.2 VLP and then with anti-GII.2 antibody. (E) Sections were incubated with GII.6 VLP and then with anti-GII.6 antibody. (F) Sections were incubated with GII.6 after incubation with the PGM-(A^+^H^+^), the flying squid liver (B^+^H^+^) and PGM (A^−^H^+^) preparations, respectively, and then with anti-GII.6 antibody. (G) Section from A blood type were incubated with (a) and without (b) GI.1 VLP and then treated with the PGM-(A^+^H^+^) followed by incubation with anti-GI.1 antibody. The section from B blood type was incubated with (c) and without (d) the VLP from GI.1 and then treated with the flying squid liver (B^+^H^+^) followed by incubation with anti-GI.1 antibody. The section from O blood type was incubated with (e) and without (f) the GII.2 VLP and then treated with the PGM (A^−^H^+^) followed by incubation with anti-GII.2 antibody. Immunostaining of all the sections from (A) to (G) was followed by an ABC detection system. See the details in the Text. Magnifications: (a), (c) and (e) in (A) to (F) and (a) to (f) in (G), ×100; (b), (d) and (f) in (A) to (F), ×400.

Binding of VLPs to human tissues was examined using resected jejunal mucosa sections from three secretor individuals with A, B and O blood types. Blood group A, B, and O(H) antigens in the mucosa from A, B and O blood types were detected by anti-A, anti-B and *Ulex* lectin, respectively (Figure 8A). The clear and contiguous positive staining from the villi to the crypts was observed at the luminal surface of the epithelia. Positive staining with *Ulex* lectin was also observed with a decreased intensity both in A and B blood type tissues corresponding to the regions stained with anti-A and anti-B (data not shown). No clear positive staining was observed in the same tissues without incubations with type specific antibodies and lectin (Figure 8B). When VLPs from GI.1 (Figure 8C), GII.2 (Figure 8D) and GII.6 (Figure 8E) were added to each jejunal tissue, positive staining was found in all the tissues from A, B and O blood types in varying small degrees. They seemed to be positively stained along with the expression of A, B and O(H) blood group substances but no significant difference was observed depending on their blood types or genotypes of the VLP added.

It was also demonstrated that nonspecific staining was absent in the same tissues without addition of each VLP (Figure 8G-b, d, f). Therefore, these binding specificities coincided well with the results from the binding assay with HGMs (Figure 2). By histochemical analyses, GI.1and GII.2 bound to the jejunal tissues of secretor individuals regardless of the A, B and O blood type. They did no show weak binding to type-B or A individuals respectively, as observed in the binding assay with saliva samples (Figure 2).

**Figure 9:**
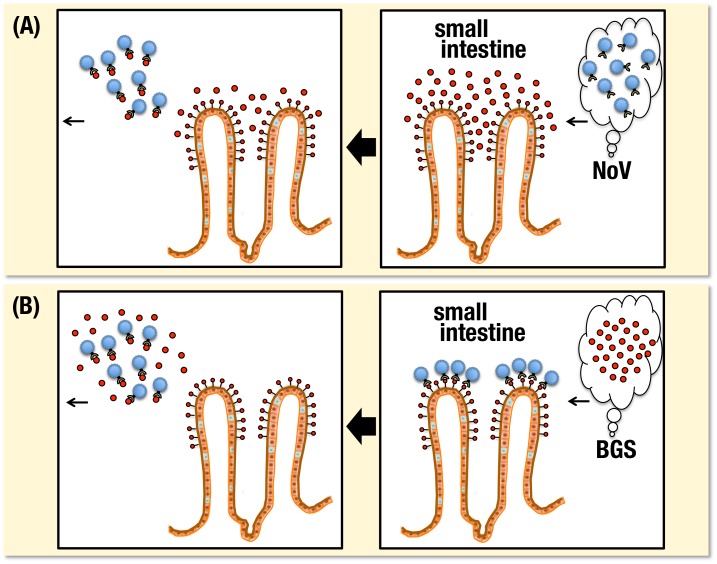
Anti-adhesion therapies aimed at prevention and treatment against NoV infection. Pre-treatment with blood group substances block NoV binding (A) and treatment with blood group substances detach NoV binding (B) to blood group-related glycans expressed on the small intestinal epithelium resulting in sweeping away of NoV infected. For treatment blood group substances are selected based on their blood group specificities corresponding to those of each NoV determined from their binding patterns to blood group related glycans.

Blocking of VLP binding to human jejunal mucosa was also examined using blood group A, B and O(H) active substances. When the VLP from GII.6, which was demonstrated to possess binding specificity to di-fucosylated and mono-fucosylated A, B and O(H) antigens in both saliva and HGM samples consistently (Figures 2,3), was pre-incubated with PGM-A^+^H^+^, TP-B^+^ and PGM-A^−^H^+^, respectively, inhibition of the staining was clearly observed (Figure 8F). Likewise, VLPs from GI.1 (Figure 8C-a,b and Figure 8C-c,d) and GII.2 (Figure 8D-e, f) bound to the tissues from A, B and O blood types, respectively, appeared largely detached by means of treatment with blood group A (Figure 8G-a), B (Figure 8G-c) and O(H) (Figure 8G-d) active substances. Therefore, results from immunohistochemical analyses of VLPs bound to jejunal mucosa with different ABO blood types showed the occurrence of blood type-specific binding, and further, it was clearly demonstrated that binding of VLPs to those tissues could be inhibited by treatment with blood group substances. For treatment blood group substances could be selected based on their blood group specificities corresponding to binding specificities of each NoV-VLP. It must be applicable from anti-adhesion therapies aimed at both prevention and treatment against NoV infection (Figure 9).

## Discussion

Antigenic determinants of blood group ABO(H) and Lewis antigens have been studied in tissues and secretions from human and animals based on immunogenetic and biomolecular analyses, and they are demonstrated to be expressed on complex glycans comprising type 1 (Galβ1,3GlcNAcβ), type 2 (Galβ1,4GlcNAcβ), type 3 (Galβ1,3GalNAcα) and type 4 (Galβ1,3GalNAcβ) chain structures attached to proteins or lipids which are present as glycoproteins or glycolipids [46], [47]. In human saliva, ABO(H) and Lewis blood group active determinants are present mainly in glycoproteins with large molecular weights secreted from salivary glands and based on the types 1, 2 and 3 chains [48], [49]. Blood group active molecules possessing the same determinants are also synthesized in and secreted from epithelium of gastric mucosa and small intestines [50], [51]. Such molecules possess heterogeneously glycosylated structures at the peripheral region on their glycans, which are synthesized by step-by-step glycosylation pathways. Thus, saliva samples from A or B and Le(a−b+) secretor individuals contain the type 1 glycans consisting of di-fucosylated (A or B/O(H)/Le^b^) and mono-fucosylated (A or B/O(H)) A or B and Le^b^ (-/Le^b^) determinants mainly, and at the same time, a set of their precursor determinants such as di-fucosylated (O(H)/Le^b^) and mono-fucosylated O(H), Le^a^ and Le^c^ determinants (Figure 1). Qualitative differences between ABO(H) and Lewis blood group and their related antigens present either in secretions such as saliva or in tissues from the digestive tract have not been clearly demonstrated yet, although intestinal mucosa may contain glycans with differently glycosylated forms depending on the development stage and differentiation state of its cells [5], [52], [53].

It has been reported that various microorganisms including viruses and bacteria recognize glycans expressed on hosts' cells and use them as a binding receptor at the time of infection [5], [54]. After Norwalk virus (NV, GI.1) was identified as the first virus associated with gastroenteritis in 1972, it was suggested that NV recognized blood group antigens expressed on the small intestine as ligands required for infection [15]. This was demonstrated with the aid of human volunteer challenge studies on NV [11], [12]. More recently, it was also found through a volunteers study conducted with a GII.4 strain [14] and further confirmed by studies of outbreaks [55]. Overall these studies together with the analysis of the glycans specificity for binding of recombinant NoV-like particles made it possible to document a relationship between genetically determined glycans expression on the gastrointestinal epithelia of hosts and susceptibility to viral infection [15], [20], [21].

A panel of saliva samples has been widely used for determination of NoV binding patterns as a conveniently procurable set of standard blood group substances with different ABO, Lewis and secretor types. Meanwhile, chemically synthesized glycans including blood group-related oligosaccharides attached to BSA could help to investigate detailed structures involved in binding epitopes. Accordingly, at least eight different binding patterns of VLPs have been reported among 16 NoV strains [56] based on the previous studies [17, 19). In the present study, binding patterns of VLPs from 16 different NoVs belonging to GI and GII genotypes were first examined using a panel of 33 saliva samples with different ABO and Lewis blood group phenotypes and secretor status including an individual with the O_h_ Bombay phenotype.

It was of particular interest that GI.11 showed a broadest binding pattern with saliva samples irrespective of their ABO and Lewis phenotypes and secretor status. Since binding of GI.11 to such saliva samples was diminished after treatments of individual saliva samples with both sodium metaperiodate (for the periodate oxidation) and several glycosidases (for the sequential removal of terminal glycosides), it suggested that GI.11 presents broad blood group-related glycan specificity. Nevertheless, binding of GI.11 VLP to gastric mucosa samples was strictly dependent upon the presence of the Lewis blood type specific fucosyl residues suggesting that subtle difference of glycans between gastric mucosa and saliva can affect recognition by some NoV stains.

Overall, eight different binding specificities were observed based on the immunological determinants synthesized on the type 1 chain structure (Table 1) in saliva samples including GI.11 and VLPs indicating inconsistent binding patterns with those reported previously. Huang *et al*. [19] reported that the VLP from OIF (GII.13) bound preferentially to saliva samples from non-secretors and that no specific binding pattern was detected with VA115 (GI.3). However, in our present study, two VLPs from the same genotypes (GII.13 and GI.3) were found to bind strongly to di-fucosylated and mono-fucosylated A, B and O(H), and di-fucosylated and mono-fucosylated A and O(H) determinants, respectively. Binding patterns different from those presented here have also been reported previously with several VLPs belonging to the same genotypes as we used [57], [58]. Structural analysis of the P2 domain from several strains of NoVs belonging to various genotypes have recently shown how minor changes in amino-acids located in the vicinity of the carbohydrate-binding site can generate modification of the carbohydrate specificity [59]–[63]. Therefore, it is possible that VLPs belonging to the same genotype may recognize different glycan structures expressed in saliva samples. Alternatively, it is also possible that genetically identical VLPs established individually by *in vitro* expression systems present micro-variations of the receptor binding interfaces and therefore that some of the reported intra-genotype differences could be artifactual.

Binding patterns of VLPs determined with HGMs seemed to be more complicated, and it was hard to identify the number of distinct binding patterns grouped based on the glycan structures to be synthesized on the type 1 chain (Figure 1) as determined with a panel of saliva samples (Table 1). Since numbers of HGM samples were limited and some of the blood types in the panel of saliva samples were not available in the binding assay with HGMs, there was no conclusive evidence, but different binding patterns observed in HGMs may be caused by the presence of either heterogeneous distributions with a set of blood group-related determinants as mentioned above, or some other molecules including glycans involved in the NoV binding. They might also be due to a higher density of glycan chains on the protein scaffold allowing to reveal interaction with minor carbohydrate epitopes otherwise unseen.

It has been widely determined that NV (GI.1) preferentially binds to the blood group O(H) and A determinants, but not to the B determinant. This binding specificity has also been ascertained in virus challenge studies from volunteers indicating that individuals belonging to B blood group have a lower risk of NV infections. Indeed, it was determined that symptomatic individuals were present only in A and O blood types but asymptomatic ones were present in B [11] and B secretor individuals [13]. However, in the present study, the VLP from GI.1 was clearly demonstrated to bind all HGMs from secretor individuals including B blood type as well as A and O type ones. The positive reactivity of the GI.1-VLP to B blood group antigen was also demonstrated in the same blood type of jejunal mucosa by immunohistochemical analyses. Further, novel binding patterns found in HGMs were repeated by immunohistochemical analyses of jejunal tissue sections with VLPs from GI.1 and GII.2, (Figure 8) as well as GII.3 , GII.7 and GII.15 (data not shown). Although the number of samples is low, so far no discrepancy of binding patterns based on the ABO blood types was found between HGM and jejunal mucosa and most importantly, specificity of the binding was ascertained through inhibition by the corresponding A, B or O(H) blood group substances. This indicates that novel binding patterns found in this study for the first time with HGMs and jejunal mucosa reflect a broad binding specificity of NoVs that include ligands of weak affinity or of weak expression, which could not be identified with a panel of saliva samples. The data of infection status obtained from volunteer studies show that such ligands (i.e., B blood group antigen and GI.1 strain) can be responsible for asymptomatic infection or a low probability of infection. Detailed analyses for structures and distributions of glycans involved in NoVs binding in the small intestine are currently under investigation using various VLPs and intestinal epithelia with different blood types through a MOLDI-TOF-MS analysis along with our recently established procedure [64].

Clear positive reactivity of VLPs with samples from non-secretor individuals was detected in around two-third of the saliva samples and in most of the HGM preparations (Figures 2,3) in this study. However, no positive binding pattern of VLPs from GI.1, GII.3 and GII.4 has been observed so far with saliva samples from non-secretors from Europe and the United States. It has already been demonstrated that non-secretor individuals of European ancestry possess the nonsense mutation at G428A in the *FUT2 (Se)* gene homozygously resulting in the lack of synthesis and secretion of ABO(H) blood group substances in secretory systems [65] but that no such mutation was found in Japanese non-secretor individuals [66]. It was also demonstrated that Japanese non-secretors possess mainly an attenuating missense mutation at A385T on the *FUT2* gene resulting in the partial suppression of ABO(H) antigens synthesis in the secretor system [67]. Therefore, positive binding reaction of VLPs found in non-secretor individuals in this study might reflect such an ethnic-associated mutation of the *FUT2* gene [68] that often results in a weak secretor phenotype rather that a true non-secretor phenotype. In fact, although no such information was available, some of the saliva and HGM samples from non-secretor individuals seemed to possess week ABH and/or Le^b^ activities (Table 2). As a result, infectivity patterns by NoVs might be slightly different between ethnic groups.

Inhibitions of NoVs binding to human saliva and gastric mucosa (HGM) samples were clearly observed in ELISA using VLPs from GI.1, GII.4 and GII.13 by addition of blood group substances prepared from porcine (PGM) and flying squid (TP) tissue extracts as well as human saliva, gastric mucosa and milk. Moreover, binding of GI.1 VLP to human saliva samples could be strongly inhibited by porcine and human gastric mucosa and human saliva samples over a wide range of concentrations (Figure 6). Blood group substances possessing the same blood types as those of the saliva and tissue samples to which VLPs bound seemed to have superior inhibiting activities compared with blood group substances possessing different blood types (Figure 7). In contrast, all the synthetic glycans except Le^Y^ attached to BSA, could hardly inhibit the GI.1 binding at their highest concentrations. This difference between natural and artificial glycans as inhibitors could be due to either the accessibility of active glycans presented in the molecules or by the co-existence of different glycans or some other molecules in HGM and jejunal mucosa, which could contribute to the VLP attachment.

At present, it seems to be hard to compare inhibiting activities of blood group substances with different blood types used in this study against binding of VLPs which have multi-binding specificities such as VLPs from GI.1, GII.4 and GII.13. It was of particular interest that PGM-H^+^ was the best inhibitor among various O(H) active blood group substances of which concentrations were adjusted by means of the anti-Fucα1,2Gal monoclonal antibody (YB-3) when inhibition assays of the GI.1 VLP binding to saliva samples possessing di-fucosylated O(H)/Le^b^, mono-fucosylated O(H) and Le^a^ determinants were conducted (Figure 6B). PGM-H^+^ might involve some active structure other than those determined immunologically by the amount of Fucα1,2Gal residues or different conformations to which the GI.1 VLP preferentially binds from other substances. Further, blood group A and B substances from PGM-A^+^H^+^ and TP-B^+^ possess O(H) activities as their precursor glycan structures on their molecules, which could also be detected by YB-3 monoclonal antibody and *Ulex* lectin, but their activities are definitely weaker than those in PGM-A^−^H^+^ (data not shown) and HGM and human saliva samples with O blood types (Table 2).

Accordingly, A, B and O(H) active blood group substances showed different activities in the plates coated with HGMs from A, B and OLe(a−b+)sec., respectively to inhibit binding of the GII.4 VLP (Figures 7B and C).

Two major binding groups of blood group related glycans for NoVs have been defined as the ABO(H) and the Lewis binding groups [8], [19] and as demonstrated in this study most of the VLPs binding to saliva and HGM samples concerned ABO(H) binding group. Therefore, a series of inhibitors to block ABO(H) type specific binding of NoVs should be promising for the development of anti-infectious agents. Blood group substances with ABO(H) activities have been reported to distribute widely in large numbers of species from lower to higher organisms [69]–[72]. Porcine tissues are considered to be good sources of blood group substances and are classified into the three phenotypes such as A^+^H^+^, A^−^H^+^, A^−^H^−^ [69]. Further, blood group B substance was isolated and purified for the first time in this study from the liver fraction of a flying squid. Accordingly, type specific inhibitions against NoVs binding to saliva samples and gastric and jejunal mucosa were clearly demonstrated using A, B and O(H) active blood group substances, respectively. Interestingly, highly efficient inhibitions were shown in both pre- and after-treatment of corresponding ABO(H) active substances against individual VLPs binding to gastric and jejunal mucosa as well as saliva samples. It suggests that blood group substances isolated from easily accessible natural sources could be used not only to block NoVs binding to the tissues but also to detach NoVs from the tissues, which might be applicable for anti-adhesion therapies both in prevention and treatment against NoV infection (Figure 9). Previously, commercially available preparations from porcine gastric mucin was reported to efficiently inhibit NV binding to human intestinal, Caco-2 cells resulting from a competitive inhibition of NV binding to the cells through the molecule expressed on the PGM [73]. Further, when the VLPs from the NV strain were pre-incubated with human milk from secretor individuals, a strong inhibition of the VLP binding to both H type 1 antigen-coated plate and human gastroduodenal tissues was clearly demonstrated and the inhibitory material from human milk was characterized in the mucin-type fraction of human milk [38], [74]. The impact of NoV gastroenteritis in infants is decreased in breast-fed children when mother's milk presents a high α1,2fucosyl/α1,3 or α1,4fucosyl residues ratio, suggesting that anti-adhesion molecules could be efficiently protective [75].

As mentioned above, two genotypes (GII.4 and GII.3) have been detected most frequently as the leading cause of non-bacterial gastroenteritis not only in Japan but also in many other countries. Nevertheless, our genetic surveillance study using a large number of specimens collected from affected children also indicated that NoVs such as GII.6 and up to 16 different GI and GII genotypes co-circulated and that at least 8 genotypes were involved in the health burden in Japan [76]. Therefore, it remains to be investigated whether blood group substances used in this study could be efficient inhibitors for binding of such NoVs as well as their variant subgenotypes to HGMs and jejunal mucosa.

Molecular surveillance studies of epidemic NoVs over the past more than one decade also indicated the emergence of new variants and recombinant subgenotypes in NoVs strains [77]-[79]. Even though it was suggested recently that continual emergence of new variants in GII.4 strains was not accompanied by a significant change of their binding specificities [80], binding assay of variant subgenotypes using human small intestinal mucosa as conducted in this study may show different binding patterns in such strains.

In conclusion, novel binding patterns were demonstrated in VLPs from several NoVs using samples of human gastric and jejunal mucosa, which were partially inconsistent with the results obtained using saliva samples possessing the same blood types. Furthermore, blood group substances with A, B and O(H) activities could be effective for preventing binding of VLPs not only to human saliva but also to human gastric and jejunal mucosa. In this endeavor, our results suggest that blood group substances prepared from edible foodstuffs including animal tissues used in this study might have potential for prevention and treatment of NoV infection (Figure 9).
