# Correction: Blood Group Substances as Potential Therapeutic Agents for the Prevention and Treatment of Infection with Noroviruses Proving Novel Binding Patterns in Human Tissues

**DOI:** 10.1371/journal.pone.0107825

**Published:** 2014-09-03

**Authors:** The PLOS ONE Staff

There are errors in the first paragraph of the Binding specificity of NoV to human saliva and gastric mucosa samples section of the Materials and Methods. The correct sentence is: VLPs from sixteen different NoVs used in this study were as follows (strain and sequence accession number); GI.1 (4656, EF547392), GI.3 (3634, EF547393), GI.4 (2876, EF547394), GI.8 (3006, EF547395), GI.11 (2258, EF547396), GII.1 (3101, EF547397), GII.2 (2840, EF547398), GII.3 (3229, EF547399), GII.4 (1207, DQ975270), GII.5 (3611, EF5473400), GII.6 (3612, EF547401), GII.7 (419, EF547402), GII.12 (2087, EF547403), GII.13 (3385, EF547404), GII.14 (2468, EF547405), and GII.15 (3625, EF547406).
